# Left Ventricle Segmentation in Echocardiography with Transformer

**DOI:** 10.3390/diagnostics13142365

**Published:** 2023-07-13

**Authors:** Minqi Liao, Yifan Lian, Yongzhao Yao, Lihua Chen, Fei Gao, Long Xu, Xin Huang, Xinxing Feng, Suxia Guo

**Affiliations:** 1 Department of Cardiology, Dongguan People’s Hospital (The Tenth Affiliated Hospital of Southern Medical Univerity), No 78, Wandao Road, Wanjiang District, Dongguan 523059, China; 2 National Space Science Center, Chinese Academy of Sciences, Beijing 100190, China; 3 University of Chinese Academy of Sciences, Beijing 100049, China; 4 National Astronomical Observatories, Chinese Academy of Sciences, Beijing 100101, China; 5 Peng Cheng National Laboratory, Shenzhen 518000, China; 6 Endocrinology Centre, Fuwai Hospital, Chinese Academy of Medical Sciences and Peking Union Medical College, Beijing 100037, China

**Keywords:** echocardiography, left ventricle, segmentation, transformer

## Abstract

Left ventricular ejection fraction (LVEF) plays as an essential role in the assessment of cardiac function, providing quantitative data support for the medical diagnosis of heart disease. Robust evaluation of the ejection fraction relies on accurate left ventricular (LV) segmentation of echocardiograms. Because human bias and expensive labor cost exist in manual echocardiographic analysis, computer algorithms of deep-learning have been developed to help human experts in segmentation tasks. Most of the previous work is based on the convolutional neural networks (CNN) structure and has achieved good results. However, the region occupied by the left ventricle is large for echocardiography. Therefore, the limited receptive field of CNN leaves much room for improvement in the effectiveness of LV segmentation. In recent years, Vision Transformer models have demonstrated their effectiveness and universality in traditional semantic segmentation tasks. Inspired by this, we propose two models that use two different pure Transformers as the basic framework for LV segmentation in echocardiography: one combines Swin Transformer and K-Net, and the other uses Segformer. We evaluate these two models on the EchoNet-Dynamic dataset of LV segmentation and compare the quantitative metrics with other models for LV segmentation. The experimental results show that the mean Dice similarity of the two models scores are 92.92% and 92.79%, respectively, which outperform most of the previous mainstream CNN models. In addition, we found that for some samples that were not easily segmented, whereas both our models successfully recognized the valve region and separated left ventricle and left atrium, the CNN model segmented them together as a single part. Therefore, it becomes possible for us to obtain accurate segmentation results through simple post-processing, by filtering out the parts with the largest circumference or pixel square. These promising results prove the effectiveness of the two models and reveal the potential of Transformer structure in echocardiographic segmentation.

## 1. Introduction

Echocardiography has become a widespread modality to get cardiac information by quickly acquiring cardiac images at a low radiation dose. Echocardiography generates spatio-temporal data in the form of short videos that can depict spatial variations in cardiac images, providing the ability to measure some dynamic motion-based diagnostic metrics, such as left ventricular ejection fraction (LVEF). The LVEF is the ratio of the difference between end-diastolic (ED) and end-systolic (ES) volumes. It is used as a quantitative metric in the diagnosis of cardiac dysfunction. If unsharp echocardiograms lead to a miscalculation of LVEF, it can delay treatment for people with heart disease, which can be fatal in many cases. Therefore, an automated and accurate assessment of LVEF is necessary.

To avoid the high-consuming human labor and enhance accuracy in echocardiogram analysis for human cardiac experts, automated algorithms for cardiac analysis have emerged. Early attempts at semantic segmentation of LV focused on formulating mathematical models based on cardiac prior knowledge. Some of these models can obtain relatively acceptable segmentation results [1]. However, most of these models were validated only on private datasets, not on large public datasets. With the success of convolutional neural networks (CNN) in semantic segmentation tasks on large datasets like ImageNet or ADE20K, deep-learning methods have become major solutions for LV segmentation in echocardiography. Multiple CNN structures like U-Net [2,3] and DeepLab [4] have been tested on LV segmentation tasks, which revealed a promising prospect of deep-learning models on 2D echocardiography. Simultaneously, larger echocardiographic datasets like EchoNet-Dynamic [4] have also been released to the public, contributing to more effective training and testing. However, in echocardiograms, every ventricle or atrium of the heart generally accounts for a large part of the pixel area, and the margins of these chambers, including heart walls and valves, are sometimes more ambiguous than normal segmentation tasks. In this case, the restricted perception field of CNN may constrain its performance in LV segmentation on echocardiograms.

Recently, Vision Transformer [5] has been widely used and has achieved excellent performance in a variety of computer vision tasks. It also has shown great potential in the field of LV segmentation in echocardiography. Deng et al. [6] proposed a network combining two CNNs connected by Transformer blocks, named TransBridge, for echocardiographic LV segmentation and achieved a Dice coefficient of 91.64%. Zeng et al. [7] developed a model named MAEF-Net for this task by fusing the channel-spatial attention mechanism on a CNN together with the efficient atrous spatial pyramid pooling (EASPP) module to collect low-resolution features. The improved results of these models compared to the CNN models suggest that these models are effective in improving the limited perceptual field of the CNN by introducing the Transformers. However, existing Transformer models on LV segmentation mainly employ the Transformer mechanism between CNN models as a subsidiary part of the CNN backbone (e.g., as a bridge structure [6]), while not make good use of its ability for feature integration. Thus, we hope to explore the advantage of Transformer models on LV segmentation. In this paper, we propose two different Transformer models to the LV segmentation in echocardiography: one combines Swin Transformer and K-Net, and the other uses Segformer. The models are evaluated on one of the largest open-sourced echocardiography datasets (i.e., **EchoNet-Dynamic**) for LV segmentation tasks on the end-diastolic frame and the end-systolic frame. We further pay attention to some samples that were not easily segmented. The segmentation performance of these samples confirms the advantages of our pure Transformer models compared to the CNN models. The major novelty of our work includes:

• Two pure Transformer automated deep-learning methods for LV segmentation in echocardiography were proposed;
• Post-processing was employed to improve some obviously missegmented results;
• The proposed methods were validated on a large public dataset (EchoNet-Dynamic [4]), with competitive performance

The organization of this paper is as follows: Some related work and their performance in echocardiographic LV segmentation are presented in Section 2. The EchoNet-Dynamic dataset, which we used to validate our model, is introduced in Section 3. The details of the two Transformer models are elaborated in Section 4. The experimental results of LV segmentation are shown and discussed in Section 5, and the conclusion is given in Section 6.

## 2. Related Work

### 2.1. Non-Deep Learning Methods

Non-deep learning segmentation models of LV in the early years focused on identifying and depicting the LV endocardium border. Methods like active contour [8] achieved relatively effective segmentation in ultrasound images, but they relied on dependence on a particular format of data with low scalability. Barbosa et al. [1] proposed a fully automated method using active contour modified by B-spline active surface, which scored 0.937 in Dice similarity on the CETUS dataset of 45 3D echocardiographic videos. Bernard et al. [9] compared nine segmentation methods (four semi-automated and five fully-automated) on a relatively fair basis by evaluation on the same dataset (RT3DE of 45 videos). The experiment proved the competitiveness of the method from Barbosa et al. [1] by providing a relatively satisfactory result. However, the results of these models were still not at the same level with expert cardiologists, and the algorithms did not demonstrate robustness on larger datasets with various modalities of LV.

### 2.2. Deep-Learning Methods

A big turnaround of LV segmentation came when deep learning was successfully deployed for extracting multi-scaled features in multiple tasks. Table 1 shows the efficiency of representative previous deep-learning models for echocardiographic LV segmentation. Suyu et al. [10] combined the traditional snake method with deep learning using a convolutional neural network for initial localization and appearance reconstruction of the snake. Smistad et al. [3] focused LV segmentation on 2D echocardiograms and successfully trained a U-Net neural network that can be well matched with the state-of-the-art automated deformable model in accuracy. Oktay et al. [11] built up a model named anatomically constrained neural network (ACNN) for 3D LV segmentation, achieving an average Dice similarity level of 0.912 (ED) and 0.873 (ES) on the CETUS dataset. In addition to U-Net, other convolutional network architectures such as ResNet [12] and DeepLab [4] have obtained promising results in experiments in this field. Simultaneously, larger 2D echocardiographic datasets with higher diversity like CAMUS [13] and EchoNet-Dynamic [4] have been used for training and testing. CAMUS provides two views (apical two-chamber and apical four-chamber) from 500 patients [13]; EchoNet Dynamic, which is used in this paper, contains 10,030 annotated echocardiogram videos [4].

Recent success of Vision Transformer (ViT) [5] promoted deep-learning based methods in computer vision by proceeding with the use of the attention mechanism. With plenty of pre-training, ViT has been proven to match the ability of classification with ResNet in the ImageNet dataset. In 2021, Liu et al. [14] proposed an efficient architecture called Swin Transformer based on ViT. By introducing attention calculation in shifted windows, Swin Transformer is efficient in extracting hierarchical features and works as the state-of-the-art backbone network on multiple tasks like object detection, image segmentation, and classification.

A few researchers have been trying to embed the attention module in the field of echocardiography. Deng et al. [6] proposed a network combined by two CNNs connected by Transformer blocks, named TransBridge, for echocardiographic LV segmentation and achieved the Dice coefficient of 91.64%. However, their process skipped the LV segmentation and could not accord with practical medical diagnosis very well. In a related field, Cao et al. [15] applied Swin Transformer blocks in the construction of U-Net architecture, proposing a U-Net-like medical image segmentor for multiple organs based on MR images. They applied the segmentor on the Automated Cardiac Diagnosis Challenge (ACDC) dataset with 100 MR images and achieved 95.83% in accuracy over R50 U-NET and Trans U-NET [16], which had already exceeded the performance of EchoNet-Dynamic in Dice. Although the model was not tested on echocardiograms, it can provide confidence for Transformer application in the medical field [15]. In addition, recent research has proven the performance of Transformer models in many fields [14,15,17]. Therefore, inspired by these studies, we consider using a pure Transformer as the model architecture for the echocardiographic LV segmentation task.

**Table 1:** Previous work of left ventricle segmentation on echocardiogram.

Bibliography	Dataset	Methods	Evaluation Metrics
[1]	CETUS	Active contour that actively fits the boundary based on maths calculation	Dice: 0.937
[2]	UCSF	CNN under traditional U-Net structure with 23 layers	IoU: 0.891
[10]	CETUS	Active snake supported by a CNN encoder as locator	modified Dice: 0.112(ED), 0.160(ES)
[3]	1500 videos	CNN based on U-Net architecture and a little training with Kalman filter	Dice: 0.870(CNN), 0.860(KF)
[11]	CETUS	CNN using auto-encoder to match the LV non-linear structure	Dice: 0.912(ED), 0.873(ES)
[4]	EchoNet-Dynamic	CNN based on Deeplab V3 architecture and atrous convolution	Dice: 0.927(ED), 0.903(ES)
[12]	CAMUS	CNN combining U-Net encoder-decoder architecture with residual blocks	Dice: 0.951
[6]	EchoNet-Dynamic	CNN encoder and decoder connected by a Transformer encoder bridge	Dice: 0.916
[16]	EchoNet-Dynamic (screened)	Transformer model based on U-Net structure for medical segmentation	Dice: 0.925
[7]	EchoNet-Dynamic (screened)	CNN embedded with channel-spatial dual attention mechanism and EASPP module	Dice: 0.931(LV)

## 3. Data

The **EchoNet-Dynamic** dataset is a large publicly available 2D echocardiogram dataset open-sourced at https://echonet.github.io/dynamic/index.html (accessed on 22 May 2023). The dataset provides 10,030 apical four-chamber (A4C) view echocardiogram videos from 10,030 individual patients. Videos of all 10,030 patients are arbitrarily divided into three subsets: TRAIN, VAL, and TEST, with 7465, 1288, and 1277 videos for model training, validation, and testing respectively.

Each echocardiogram in **EchoNet-Dynamic** has been processed to be a 112 × 112 × 3 beat-to-beat clip containing end-systole (ES) and end-diastolic (ED) frames. With several beats included in each video, one frame each for ES and ED is selected for calculating end-systole volume (ESV) and end-diastolic volume (EDV). Based on these symbolic frames, expert tracings are given in the form of pairs of coordinates, which can depict the volume and shape of the LV from two axes. We take these symbolic frames from expert tracings as the inputs of our models introduced in the next section.

**Figure 1:**
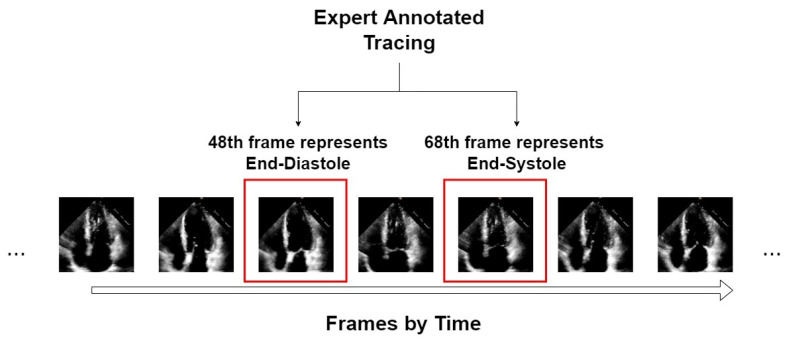
An example of an echocardiogram from the EchoNet-Dynamic dataset.

An example of the form of data from the EchoNet-Dynamic dataset is shown in Figure 1. We take video “0X1A05DFFFCAFB253B” as an example, which is about a 3 s video with 50 frames in a second. From expert tracing, the algorithm can accurately position the 48th frame that represents ED and the 68th frame that represents ES. For this video, only these two symbolic frames are extracted as the input of our segmentors.

## 4. Methodology

This section will elaborate on the two recent Transformer-based networks we experimented with in this paper. With the Transformer raised in the NLP field to modify the encoder–decoder structure initially [18], major followers of Transformer have been proposing modifications on their encoder–decoder networks [6,9,17] by embedding the Transformer module. Witnessing their high efficiency in segmentation tasks on universal datasets, like ImageNet and ADE20K, two encoder–decoder networks, **Swin Transformer and K-Net** and **Segformer Network**, are introduced to echocardiographic LV segmentation.

For the multi-task application in a general dataset, Zhang et al. [19] designed the K-Net with an iterative decode head for multiple choices of backbones including CNN and Transformer. Combined with Swin Transformer blocks, K-Net can get benchmark results compared with using other backbones on multiple tasks.

In addition to Swin Transformer, Xie et al. [17] proposed another improvement on ViT, namely Segformer, which is an encoder–decoder network combined with an improved ViT Encoder (MiT) and a lightweight MLP decoder. As an integrated network, the Segformer scored better on ADE20K and Cityscapes than PSPNet, DeepLab, and SETR [17].

**Figure 2:**
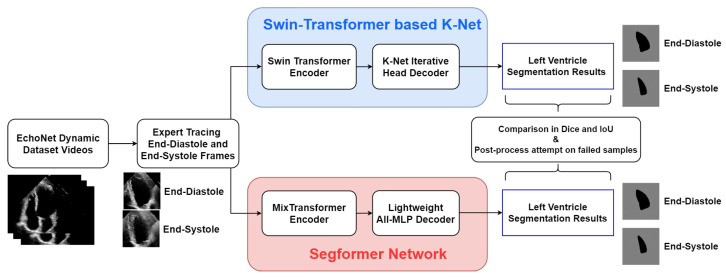
Overall framework of the research in this study.

Figure 2 shows the process framework of the research presented in this paper. For both of the two networks, the EchoNet-Dynamic dataset extracts representative frames of end-systole and end-diastole. These two frames of every echocardiogram are labelled in the expert tracings and extracted as the input of every video. The two encoder–decoder networks process these frames and finally output the LV segmentation results in the form of pictures. Simultaneously, indexes of accuracy are calculated from comparing expert tracing results and algorithmic results to show the performance of the two models.

### 4.1. Swin Transformer and K-Net

**Figure 3:**
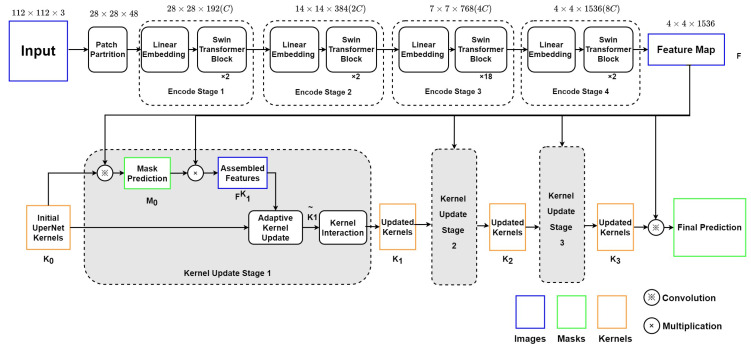
Structure of Swin Transformer and K-Net.

As depicted in Figure 3, this encoder–decoder model includes two main modules: (1) the Swin Transformer backbone, which can collect and encode multi-level features with less computational complexity; (2) a K-Net network using iterative decode head to provide the semantic segmentation result on the left ventricle.

#### 4.1.1. Swin Transformer Blocks as Encoder

The Swin Transformer block will split the echocardiography image into 784 patches of 4 × 4 pixels. Each patch has a feature dimension of 4 × 4 × 3 = 48 and is taken as a “token”. In Stage 1 of the encoder, these patches with original features are projected into an arbitrary dimension (*C*) by the linear embedding layer. The arbitrary dimension (*C*) represents the capacity of information embedded in each token. With the Swin-Large config, *C* is set to 192. Compared with ViT [5], whose dimension is fixed as 768 for each token, Swin Transformer utilizes a smaller initial pixel region with fewer channels, although, through stages, the number of channels increases with patch merging. Swin Transformer blocks will be processed on these patches to compute the attention. Each pair of Swin Transformer blocks computes the attention among patches within the *M* × *M* window and the shifted window. Stage 1 finishes with 784 tokens under the *C* dimension, as the Swin Transformer blocks do not change the number and dimension of tokens.

Then, in Stage 2, the adjacent patches are concatenated by the patch merging layer for hierarchical feature collection. This will simultaneously double the dimension to 2C and reduce the tokens to 196. Two Swin Transformation blocks will then compute the window-attention among these larger tokens. This process, consisting of the patch merging layer and several Swin Transformer blocks, repeats two more times as ‘Stage 3’ and ‘Stage 4’.

The Swin Transformer encoder finally outputs a 4 × 4 × 1536 tensor as the input feature map *F* for the decode head.

#### 4.1.2. K-Net Iterative Decode Head as Decoder

K-Net provides a solution of decode head iteration to get more accurate results in semantic segmentation. Initial kernel $K_{0}$ is chosen to be the UPerNet decode head [20] in our research. Before every stage *n* of iteration, kernels $K_{n-1}$ produce initial mask production $M_{n-1}$ by convolution with the input feature map *F*.

Then, the *Kernel Update Head* begins with the multiplication of *F* and $M_{n-1}$ to get assembled feature $F_{n}^{K}$. The produced $F_{n}^{K}$ could represent the customized features towards each segmentation object for the input kernels in the *n*th round.

In the second step, the element-wise product of $F_{n}^{K}$ and $K_{0}$ produces $F_{n}^{G}$ for gate calculation. Then, $F_{n}^{G}$ is used to calculate two gates, $G_{n}^{F}$ and $G_{n}^{K}$, to represent the proportion of $F_{n}^{K}$ and $K_{n-1}$ in the updated kernel ${\tilde{K}}_{n}$.

The third step focuses on the contextual information integration into the updated kernels. A feed-forward neural network is used to compute the multi-head attention of kernels ${\tilde{K}}_{n}$, and the output $K_{n}$ is used to produce a new mask $M_{n}$ or the final prediction (*n* = 3).

### 4.2. Segfomer Network

**Figure 4:**
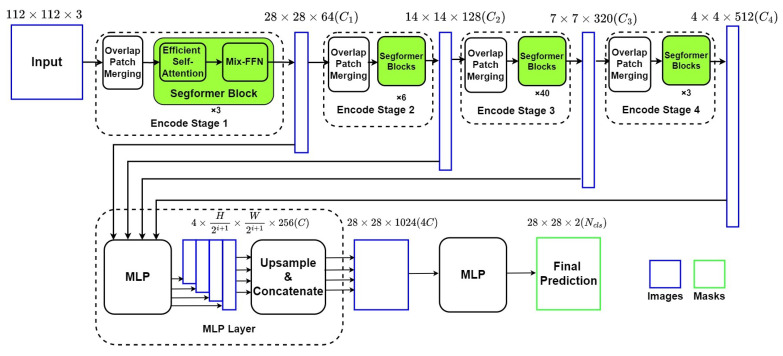
Structure of Segformer Network.

Figure 4 describes the framework of the **Segformer** network, which is also an encoder–decoder network with two main components: (1) a Mix Vision Transformer (MiT) which is a modified Vision Transformer based on Mix-FFN instead of positioning code; (2) a lightweight MLP decoder, which can integrate local and global attention with less computing complexity.

#### 4.2.1. Mixed Vision Transformer as Encoder

The MiT Encoder includes four stages with the *Overlapped Patch Merging Layer* and the *Transformer Block*. The *Overlapped Patch Merging Layer* reshapes the feature map at the start of every stage by a convolutional neural network, which makes the feature map become a quarter in Stage 1 and a half in Stages 2–4. In terms of dimension, this reshape in Stage *i* also projects the feature map to the corresponding specified dimension $C_{i}$. Thus, the hierarchical feature maps are obtained by concatenating feature maps produced by all four stages in the decoder.

The *Transformer Block* in MiT includes numbers of combinations of modified self-attention and improved position encoder *Mix-FFN*. To overcome the bottleneck of self-attention calculation in ViT, MiT adds a reshape factor *R* to transform the factor *K* from *N* × *C* (*N* = *H* × *W*) into $\frac{N}{R}$ × *C*. The position encoder *Mix-FFN* consists of MLP layers, a 3 × 3 convolutional layer, and a GELU activation function, which can be expressed as:

(1) $$x_{out}=MLP\left(GELU\left(Conv_{3\times 3}\left(MLP\left(x_{in}\right)\right)\right)\right)+x_{in}$$

Noted that the GELU function is widely used in Transformer models such as ViT [5] and can resolve the gradient disappearance caused by negative inputs. The output of each *Mix-FFN* layer will form a list as the output of MiT network.

#### 4.2.2. The Lightweight Segformer Decoder

Based on the relatively larger effective receptive field, Segformer is equipped with a lightweight decoder with only MLP layers. Output from every MiT Stage is reshaped from $\frac{H}{2^{i+1}}$ × $\frac{W}{2^{i+1}}$ × *C* (*i* as the layer number) into $\frac{H}{4}$ × $\frac{W}{4}$ × *C* through a MLP layer and an upsampling layer. Then the four tensors are concatenated into a $\frac{H}{4}$ × $\frac{W}{4}$ × *4C*, which works as the input of the final MLP layer for final segmentation. In this paper, *C* is set to 256, and the final segmentation result is divided to two groups: ventricle and background, as we are only interested in segmentation of LV.

## 5. Experiments and Results

### 5.1. Implementation Details

As introduced in Section 3, the EchoNet-Dynamic datasets split the 10,030 echocardiograms into three groups: TRAIN, VAL, and TEST, with 7465, 1288, and 1277 videos, respectively. We followed this split in our experiment, using the TRAIN set for training, the VAL set for validation after every 5 epochs of training, and the TEST set for the final test.

As for the evaluation metrics, we use Dice coefficient index (Dice) to compare our performance with previous work [4,6]. We also use intersection over union (IoU) for more comprehensive comparison. These metrics are calculated based on the predicted LV region (*S*) and ground truth of human expert segmentation results from the EchoNet-Dynamic dataset ($S_{E}$). The equations of IoU (2) and Dice (3) are shown below:

(2) $$IoU=\frac{S\cap S_{E}}{S\cup S_{E}}$$

(3) $$Dice=\frac{2(S\cap S_{E})}{S+S_{E}}$$

To optimize the training process, we use ADE20K pre-trained weights for both of the two models. The initial learning rate is set to 6e-5 for both models. During the training process, the AdamW optimizer is implemented to improve the effect of the cross-entropy loss function. In terms of computing environment, both models are trained on an NVIDIA RTX3060 GPU for 50 epochs.

### 5.2. Experimental Results

**Figure 5:**
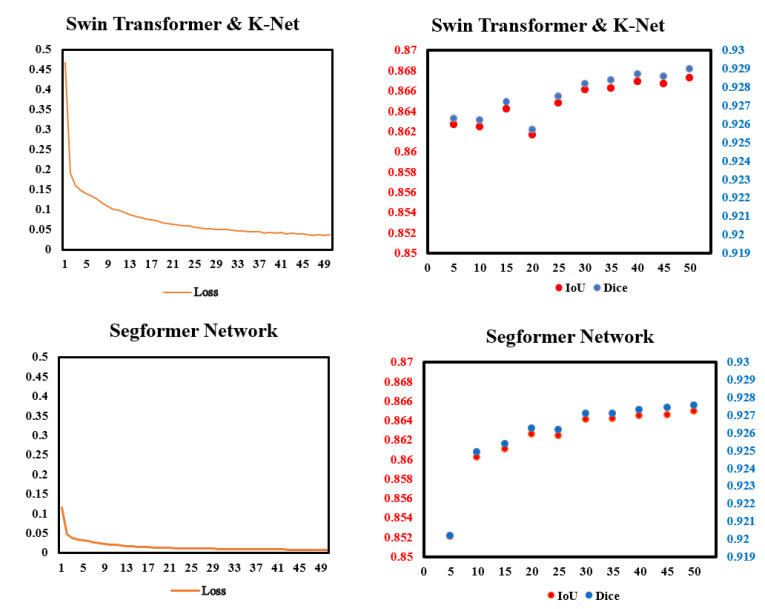
Data monitor of the two models in this paper in training and validation. (**Left**): Pixel accuracy (%) and loss in the training process of the two models. (**Right**): IoU and Dice in the validation of every 5 epochs.

To check whether the models work properly, we monitored the loss in the training process and the IoU and Dice in every validation. Figure 5 shows data we monitored. In the training process, the improvements of loss was more significant in Swin Transformer and K-Net than in Segformer. In validation, the performances of Dice and IoU of the two models gradually became steady after 30 epochs of training. For both IoU and Dice scores, Swin Transformer and K-Net performs slightly better than Segformer in most validations.

In Table 2, the statistics of IoU and Dice of LV segmentation for the TEST subset of the EchoNet-Dynamic dataset are shown. For comparison, EchoNet-Dynamic Network [4] and TransBridge [6] are taken as the benchmarks of previous work, with the former as a traditional CNN model and the latter as a Transformer-embedded model. Among these models, the two Transformer models in our research achieved the best Dice, with 92.92% and 92.79%, respectively. Compared with the EchoNet-Dynamic Network [4]%, the Dice of Swin Transformer and K-Net is higher by 1.42%, and for the Transformer-embedded model [6], the gap in Dice is 1.28%. Furthermore, among the two models, Swin Transformer shows better performance in LV segmentation than Segformer, regardless of whether from the perspective of IoU or Dice.

**Figure 6:**
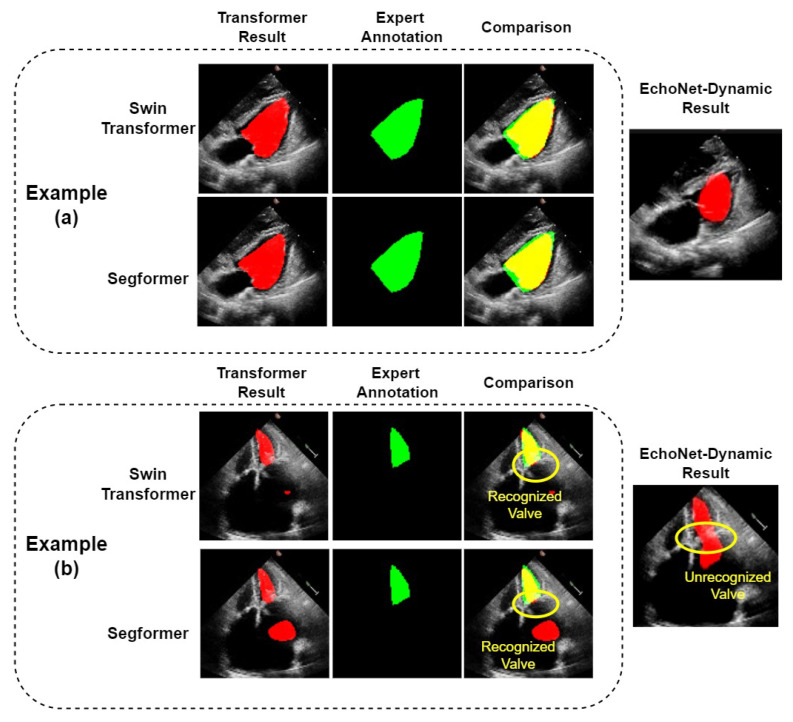
Comparison of LV segmentation between the two Transformers. (**a**): A normal example where Transformer models have better performance, as the outline of the ventricle is better segmented; (**b**): a special example where all the models fail, although the Transformer models we use successfully recognize the valve and segmented the ventricle and atrium.

The two Transformer models show good performance on most echocardiograms; on some of which they obviously outperformed the CNN model, EchoNet-Dynamic, as shown in example (a) of Figure 6. However, we found in our experiments that our model also makes significant segmentation errors in a few samples. For these samples, the LVs segmented by our models differ from their corresponding real ones. Example (b) of Figure 6 shows an example in which both the Swin Transformer and Segformer models failed, as they mistakenly treated some part of the left atrium as the left ventricle. It can be seen that the same error occurs with the EchoNet-Dynamic model. However, compared with EchoNet-Dynamic results, both of our models correctly divide the left ventricle and left atrium into two parts. We can obtain the accurate results by simple post-processing. The post-processing can recognize the part with the longest perimeter in the predicted region and remove other parts, including the misidentified part in the left atrium. To avoid affecting the performance of normal segmentation results, we only use post-processing on those with at least two segmented parts. As shown in Table 2, the IoU of Swin Transformer and K-Net with post-processing and Segformer Network with post-processing are 86.78% and 86.57%, respectively. The Dice of these two models with post-processing are 92.92% and 92.80%, respectively. Compared with the results of their corresponding models without post-processing, both the results of IoU and Dice are the same or slightly better. The reason should be that the small number of such samples makes the improvement in the overall metrics insignificant.

**Figure 7:**
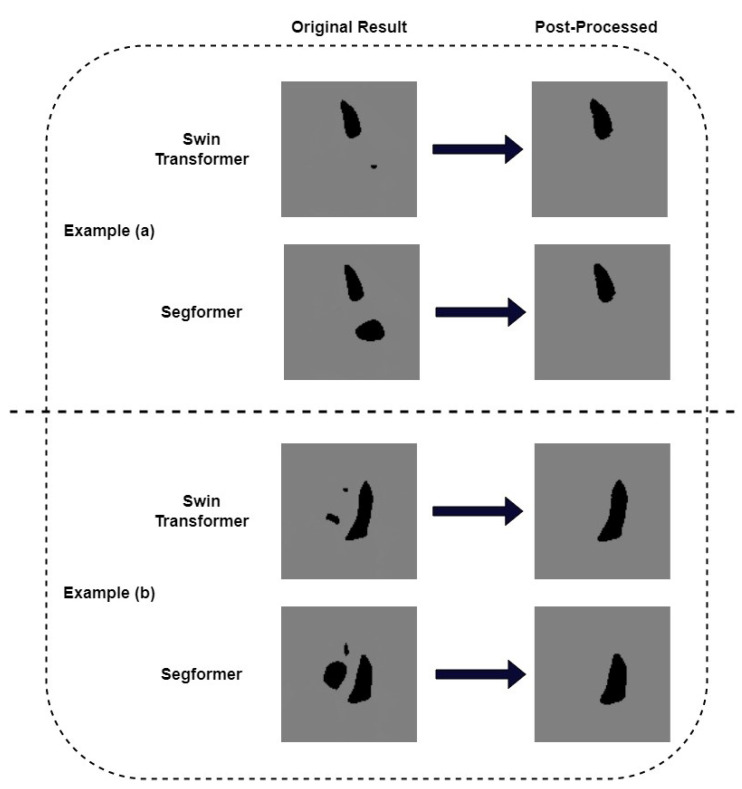
Post-process by recognizing largest perimeter on the seriously failed results. (**a**) an example where post-process corrected the mis-segmented left atrium (**b**) another example where post-process corrected the mis-segmented right ventricle and right atrium.

We pay further attention to the mis-segmented samples. Figure 7 shows two examples where we successfully removed the wrongly recognized parts by post-processing. It can be seen that the results obtained after post-processing are consistent with the ground truth. In order to quantitatively measure the correlation between post-processed segmentation results and the ground truth, we choose example (b) in Figure 6 as a typical sample and calculate its metrics. Table 3 shows the IoU and Dice of the missegmented sample of our models. The IoU of Swin Transformer and K-Net and its corresponding model with post-processing are 84.82% and 86.79%, respectively. The Dice of these two models are 91.79% and 92.93%, respectively. For the Segformer model and its corresponding model with post-processing, their IoUs are 73.19% and 82.11%, respectively. The Dice values are 84.52% and 90.18%, respectively. It can be seen that, for this sample, both IoU and Dice are much improved after post-processing. Although both metrics of the Segformer model improved more, the results of the Swin Transformer and K-Net model were higher. We also calculated the Dice of the EchoNet-Dynamic model on this missegmented sample as a comparison. The Dice of this model is 63.97%. The results above show that our proposed Transformer models presented an alternative method for segmentation in echocardiography, with a little improvement in Dice similarity. It is worth noting that our Transformer models allow for some simple post-processing to improve some serious segmentation errors, which previous CNN models cannot do.

## 6. Conclusions and Discussion

In this paper, we propose two models that use two different pure Transformer models as the basic framework for LV segmentation in echocardiography: one combines Swin Transformer and K-Net, and the other uses Segformer. We focused on just end-systolic frames and end-diastolic frames, which are used for LVEF calculation. Our models are evaluated on the the EchoNet-Dynamic dataset.

From the quantitative experimental results, the proposed two models outperform most of the previous models, including traditional DeepLab v3+ [4], the TransBridge model [6], and the Trans U-Net model [7]. The Dice of the Swin Transformer and K-Net and Segformer are 92.92% and 92.79%, respectively. These two models validated with good applicability in echocardiographic LV segmentation, especially the Swin Transformer and K-Net model. In addition to the overall quantitative metrics, we focused on some samples that were not easily segmented that could be even more important in practical medical applications. The results show that, for these samples, although our models did not accurately segment the left ventricle, both of them could successfully recognized the boundary of the LV like the cardiac valve and separate the main ventricle area with other cardiac parts (e.g., left atrium), to avoid segmenting them into one unit. In these cases, we can obtain accurate segmentation results through simple post-processing. This is difficult to achieve for CNN models because of their limited receptive field. Our model differs from previous work by applying a pure Transformer architecture for the LV segmentation task. Existing LV segmentation models using Transformer either introduce attention mechanisms into CNN or combine the Transformer blocks with CNN structure. However, the region occupied by the left ventricle is too large for echocardiography. The limited receptive field of CNN makes it less suitable than Transformer for LV segmentation tasks. The experimental results demonstrate the effectiveness of our models.

This work configured two alternative Transformer methods for LV segmentation in echocardiography, which show competitive performance compared with traditional CNN methods. This could, in some way, reveal the potential of Transformers in clinical applications. Furthermore, simple post-processing is effectively validated to significantly improve some results with serious errors. We hope this can provide some inspiration for the development of automated algorithms in echocardiographic analysis.

There are several limitations in this work. First, we just focused on the static frames of ED and ES, ignoring the dynamic periods in each heartbeat. As practically applicable functions like ED/ES frame detection relies on the video-based algorithm, it could be necessary for validation on video. Second, during the examination of our results, we found that there are several mis-traced samples in the EchoNet-Dynamic dataset. To some extent, this could affect the final performance of our models, although for a parallel comparison with a former study, we hold the results with these samples. In addition, this paper has not addressed the automated calculation of LVEF, which is included in normal clinical processes. In future work, we will integrate the calculation of LVEF, which also provides another metric for performance evaluation.

Therefore, we intend to propose models capable of directly performing LV segmentation on echocardiographic videos in future work. 

## Figures and Tables

**Table 2:** Comparison of different models on LV segmentation on EchoNet-Dynamic dataset.

Methods	IoU%	Dice%
EchoNet-Dynamic Network [4]	-	91.50
TransBridge [6]	-	91.64
Trans U-net [16]	-	92.54
**Swin Transformer and K-Net**	**86.78**	**92.92**
**Swin Transformer and K-Net with post-processing**	**86.78**	**92.92**
**Segformer Network**	86.56	92.79
**Segformer Network with post-processing**	86.57	92.80

**Table 3:** Metrics of representative missegmented samples after post-processing of the two Transformer models.

Metrics	EchoNet-Dynamic [4]	Swin Transformer and K-Net	With Post-Processing	Segformer	With Post-Processing
IoU %	-	84.82	86.79	73.19	82.11
Dice %	63.97	91.79	92.93	84.52	90.18

## Data Availability

Not applicable.

## Abbreviations

The following abbreviations are used in this paper:

LV	Left ventricle
LVEF	Left ventricle ejection fraction
ED	End diastole (End-diastolic)
ES	End systole (End-systolic)
EDV	End-diastole volume
ESV	End-systole volume
ViT	Vision Transformer
MIT	Mixed Vision Transformer
